# Different effects of allergic rhinitis on nasal mucosa remodeling in chronic rhinosinusitis with and without nasal polyps

**DOI:** 10.1007/s00405-018-5195-x

**Published:** 2018-11-16

**Authors:** Rong Xiang, Qing-ping Zhang, Wei Zhang, Yong-gang Kong, Lu Tan, Shi-ming Chen, Yu-Qin Deng, Ze-zhang Tao, Yu Xu

**Affiliations:** 1 Department of Otorhinolaryngology Head and Neck Surgery, Renmin Hospital of Wuhan University, 238 Jiefang Rd, Wuhan, 430060 Hubei People’s Republic of China; 2 Department of Otorhinolaryngology Head and Neck Surgery, Suizhou Hospital, Hubei University of Medicine, 60 Longmen Rd, Suizhou, 441300 Hubei People’s Republic of China

**Keywords:** Chronic rhinosinusitis, Allergic rhinitis, Nasal polyp, Remodeling

## Abstract

**Background:**

Allergic rhinitis (AR) has been reported to be associated with chronic rhinosinusitis (CRS). The objective of this study was to investigate the effect of AR on nasal mucosa remodeling in CRS.

**Methods:**

Patients were enrolled and divided into the following groups: CRS with nasal polyps (NP) with allergic rhinitis (AR)(CRSwNPwAR; *n* = 20), CRS with NP without AR (CRSwNPsAR; *n* = 20), CRS without NP with AR (CRSsNPwAR; *n* = 20), CRS without NP without AR (CRSsNPsAR; *n* = 20), AR without CRS (AR; *n* = 20) and controls (*n* = 14). Eosinophil infiltration, mucus production, and collagen deposition were examined by hematoxylin and eosin, periodic acid schiff and masson’s trichrome staining, respectively. VEGF-A and microvessel density were detected by immunohistochemistry. The expression of remodeling markers, including TGF-β1, MMP-7, MMP-9 and TIMP-1 were measured by Western blot.

**Results:**

The expression of remodeling factors, including VEGF-A, CD31, CD34 and TIMP-1 were significantly increased in CRSwAR compared to CRSsAR. Goblet cell hyperplasia, as well as VEGF-A, CD31, CD34, and MMP-9 expression were significantly higher in CRSwNPwAR compared to CRSwNPsAR. However, the expression of collagen fibers, MMP-7 and TGF-β1 were significantly higher in CRSsNPwAR compared to CRSsNPsAR.

**Conclusions:**

AR could enhance the remodeling process in CRS. Moreover, AR had different effects on CRSwNP and CRSsNP.

## Introduction

Chronic rhinosinusitis (CRS) is a chronic inflammation of the nasal cavity and paranasal sinus mucosa that affects 12.5% of the population worldwide [1]. CRS is accompanied by symptoms, including nasal discharge, nasal blockage, loss of smell and headache. It causes a significant socioeconomic burden on health care system and severely impacts patients’ quality of life [2]. CRS can be divided into two phenotypes, based on the presence or absence of nasal polyps, referred to as chronic rhinosinusitis with nasal polyps (CRSwNP) and chronic rhinosinusitis without nasal polyps (CRSsNP), respectively [3]. Different phenotypes of CRS have different immunological mechanisms and different remodeling features as well. CRSwNP is a Th2-skewed response with high levels of IL-4, IL-13 and IL-5, and an eosinophil (EOS) predominance [4]. It is characterized by pseudocysts formation, lack of collagen within the extracellular matrix and lower expressed TGF-β1. Conversely, CRSsNP is Th1-skewed neutrophilic inflammation with high levels of IFN-γ, TGF-β1 and subsequently excessive collagen deposition [5].

The goals of treatment for CRS are to achieve a local disease control and reduce patients’ symptoms. Generally, conservative therapies, including topical application of steroids and nasal saline irrigations, are applied to patients with CRS to reduce underlying sinonasal inflammation. Surgical interventions, such as endoscopic sinus surgery, are reserved to improve sinus ventilation and mucociliary clearance for patients who fail maximal medical therapy. However, the current therapies for patients with CRS are unsatisfactory. It was estimated that up to 20% of CRS patients were not well-controlled by surgical interventions and required revision during the 5-year follow-up [6]. Apart from surgical skills, cystic fibrosis, immune deficiencies, primary ciliary dyskinesia, smoking and asthma, allergic rhinitis (AR) has also been reported to negatively affect the outcome of CRS treatment [7]. Evidence suggests that AR is an inflammatory instigator or an exacerbating factor for CRS. Among pediatric patients with CRS, 36–60% have been diagnosed with AR [8]. And, the patients who underwent functional endoscopic sinus surgery takes significantly longer time to recover from the surgery if they had a history of AR compared to those who did not [9]. Besides, the symptoms scores of CRS patients with AR (CRSwAR) are generally significantly improved in patients treated with immunotherapy compared to baseline data and control patients [10]. Although some studies had investigated the association between AR and CRS, little is known about the mechanisms by which AR affects the progression of CRS.

Currently, evidence suggested a relationship between inflammation and remodeling [3, 11]. Lots of inflammatory mediators are reported to play important roles in both inflammation and remodeling processes [12, 13]. For instance, TGF-β1 could inhibit T-cell activation and initiate persistent epithelia activation and structural remodeling at the same time [12]. Eosinophils (EOS) inflammation was associated with edema formation in CRS [12]. Considering the close relationship between inflammation and remodeling, we hypothesized that AR, as a chronic inflammatory disease, could affect the pathogenesis of CRS by regulating remodeling progress in the nasal mucosa.

## Methods

### Subjects and samples

This study was performed on patients who underwent endoscopic nasal surgery at the Department of Otorhinolaryngology-Head and Neck Surgery of the Renmin Hospital of Wuhan University (Wuhan, China) from January 2015 to December 2016. Patients were separated into the following six groups: CRSwNP with AR (CRSwNPwAR; *n* = 20), CRSwNP without AR (CRSwNPsAR; *n* = 20), CRSsNP with AR (CRSsNPwAR; *n* = 20), CRSsNP without AR (CRSsNPsAR; *n* = 20), AR without CRS (*n* = 20) and patients with only anatomic variations without sinus diseases as controls (*n* = 14). This study was approved by the Ethics Committee of Renmin Hospital of Wuhan University (No. WDRY2016-K002). Informed consent was obtained from every subject.

The diagnosis of CRS in each patients was based on patients’ symptoms, nasal endoscopy examination, and sinus computed tomography (CT) scan in accordance with the recommended European diagnostic standard EPOS2012 [14]. The diagnosis of AR was based on the presence of nasal symptoms, positive skin prick test, and serum IgE levels according to the Allergic Rhinitis and its Impact on Asthma (ARIA) guidelines [15]. Patients with CRSwNPsAR or CRSsNPsAR or controls had negative skin prick tests. All patients underwent CT examination for Lund-Mackay scoring and nasal endoscopy for Lund-Kennedy (LK) scoring. Visual analog scale (VAS) scores and 20-item Sino-Nasal Outcome Test (SNOT-20) were done for each symptom for all patients. Other information on asthma, history of endoscopic sinus surgery and smoking was collected at the same time. Patients were excluded if they had a history of autoimmune disease, the ‘aspirin triad’, primary cilia motility dysfunction or cystic fibrosis, or had a history of intranasal or oral corticosteroid or decongestants and antihistamines use in the 4 weeks prior to the surgery.

Nasal polyps tissues in patients with CRSwNPwAR or CRSwNPsAR were collected during the surgery. Uncinate process mucosa was collected from patients with CRSsNPwAR or CRSsNPsAR. The uncinate process mucosa of AR group was taken from patients underwent septoplasty. The inferior turbinate mucosa was collected from control patients who underwent septoplasty in the absence of sinus diseases and allergic disease. Tissues were collected at the time of surgery and divided into two portions. One was reserved in liquid nitrogen for protein extraction, while the other was fixed in 4% paraformaldehyde for 24 h prior to embedding in paraffin.

### Paraffin section staining

The paraffin-embedded tissues were sectioned at a thickness of 5 µm for hematoxylin and eosin (HE), periodic acid Schiff (PAS) and Masson’s trichrome staining. All stains were performed in accordance with the manufacturer’s protocol. HE staining was performed to examine EOS infiltration in lamina propria and basement membrane thickness. Five randomly selected fields in each slice were analyzed with a constant mucosal length under a high-power field (× 400) by two separate investigators. PAS staining was used to evaluate mucus production in epithelia and glands. Positive-goblet cells were calculated in five randomly selected fields in each sample at 200× magnifications. Masson’s trichrome staining was performed to assess collagen deposition. Collagen volume fraction was quantified in five randomly selected fields at 200×magnifications using Image Pro Plus 6.0 (Media Cybernetics, Silver Spring, MD, USA).

### Western blotting

The tissues were cut into pieces and the total protein was extracted by RIPA lyses buffer (Beyotime, China). The total protein was quantitated by QuantiPro BCA assay kit (Sigma, St. Louise, MO, USA) according to the manufacturer’s instructions. Protein samples (25 µg per lane) were separated by 10% SDS–PAGE gel and subsequently transferred onto polyvinylidene difluoride (PVDF) membranes. The membranes were blocked for 2 h at room temperature in TBST containing 5% skim milk. After 5 min washing in TBS, the membranes were subsequently incubated with monoclonal rabbit anti-human MMP7 primary antibody (1:1000; cat no. ab207299; Abcam, Cambridge, MA, USA), monoclonal rabbit anti-human MMP9 primary antibody (1:1000; cat no. ab137867; Abcam, Cambridge, MA, USA), monoclonal rabbit anti-human TIMP1 primary antibody (1:1000; cat no. ab211926; Abcam, Cambridge, MA, USA), polyclonal rabbit anti-human TGF-β1 primary antibody (1:1000; cat. no. ab92486; Abcam, Cambridge, MA, USA) or monoclonal rabbit anti-human β-actin primary antibody (1:1000; cat. no. ab109499; Abcam) at 4 °C overnight. Following extensive washing in TBST, the membranes were incubated with polyclonal goat anti-rabbit secondary antibody conjugated with horseradish peroxidase (1:1,000; cat. no. ab6721; Abcam) for 1 h at room temperature. Immunoreactive bands were detected by the electrochemiluminescence Western blotting detection system (LI-Cor Biosciences, Lincoln, NE).

### Immunohistochemistry

Immunohistochemistry (IHC) procedure was performed using the streptavidin biotin complex (SABC) kit (Boster Biological Technology). Briefly, after deparaffinization and rehydration, the tissue sections were microwaved for 10 min in citrate buffer for antigen retrieval and then incubated separately with primary rabbit polyclonal antibody against CD31 (1:100; cat. no. ab28364; Abcam) and primary rabbit polyclonal antibody against CD34 (1:100; cat. no. ab110643; Abcam) overnight at 4 °C to stain target protein expression. Following 20-min incubation with secondary anti-rabbit antibody, 3,3-diaminobenzidine (DAB) was applied for visualizing immunoreactivity. Five randomly selected fields in each slice were quantified and the integrated optical density (IOD) immunoreactivity was calculated as the relative products of the stained area using Image Pro Plus 6.0 (Media Cybernetics, Silver Spring, MD, USA).

### Statistical analysis

Experimental results were expressed as mean ± SD. Data analysis was conducted using SPSS software (version 17.0; SPSS, Inc., Chicago, IL, USA). Clinical characteristics were compared among groups using Chi square test. One-way ANOVA with Tukey analysis was performed to compare variabilities among groups. A level of *p* < 0.05 was regarded as statistically significant.

## Results

### Patient characteristics

The clinical characteristics summarized for all patients are shown in Table 1. There was no significant difference among the six groups with respect to gender and age distribution (*p* > 0.05). The LK endoscopy scores were significantly higher in the CRSwNPwAR group compared to the CRSsNPwAR group.

**Table 1:** Characteristics of the included subjects

Characteristic	Control	AR	CRSwNPwAR	CRSsNPwAR	CRSwNPsAR	CRSsNPsAR	*p*
Number of patients (male/female)	14 (8/6)	20 (12/8)	20 (9/11)	20 (10/10)	20 (7/13)	20 (9/11)	*p* > 0.05
Median age, years (IQR)	29.9 (19–36)	39.5 (25–68)	37.3 (22–65)	47.2 (25–74)	40.2 (18–68)	34.6 (20–68)	*p* > 0.05
Surgery, *n*	0	0	5	3	2	0	
History of smoking, *n*	5	12	10	8	9	7	
History of asthma, *n*	0	2	4	2	0	0	
Uni-VAS	7.43 ± 1.77	12.33 ± 1.78	31.45 ± 4.5	29.56 ± 3.8	28.9 ± 14.7	30.9 ± 1.89	*p* > 0.05
Lund Kennedy endoscopy score	4 ± 1.22	5.0 ± 1.2	11.34 ± 1.27	4.8 ± 1.45	9.35 ± 2.24	8.37 ± 1.9	*p* < 0.01
Lund-Mackay CT score	3 ± 0.2	5.0 ± 1.7	20.89 ± 8.9	16.77 ± 4.88	17.44 ± 7.8	18.0 ± 5.2	*p* > 0.05
SNOT-20 score	5.76 ± 1.23	12.31 ± 2.14	27.45 ± 2.7	28.56 ± 3.4	26.67 ± 3.8	24.55 ± 6.2	*p* > 0.05

Comparison of the gender and number of patients was performed using the Chi square test. One-way ANOVA was applied to calculate the significance differences of Uni-VAS, LK endscopy score, LM CT score and SNOT-20 score among CRSwNPwAR, CRSwNPsAR, CRSsNPwAR and CRSsNPsAR groups.

AR allergic rhinitis, CRSwNPwAR chronic rhinosinusitis with nasal polyps with allergic rhinitis, CRSsNPwAR chronic rhinosinusitis without nasal polyps with allergic rhinitis, CRSwNPsAR chronic rhinosinusitis with nasal polyps without allergic rhinitis, CRSsNPsAR chronic rhinosinusitis without nasal polyps without allergic rhinitis, VAS visual analogue scale, CT computed tomography, SNOT-20 20-item sino-nasal outcome test

### Histological analysis

**Fig. 1:**
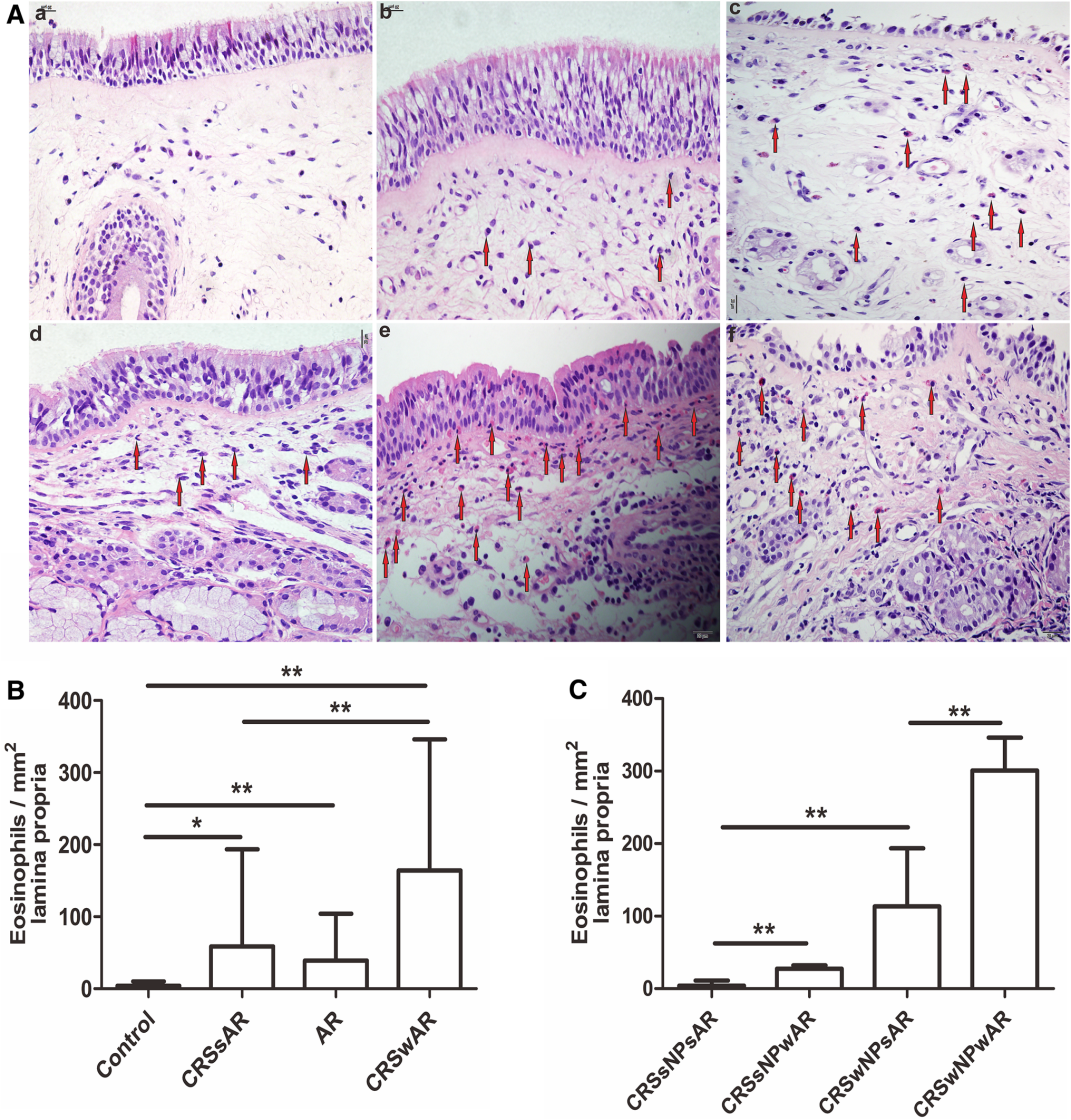
**A** Infiltration of eosinophils in lamina propria was examined by HE staining. **B**, **C** Quantification of EOS in the nasal mucosa.** a** control group,** b** AR group,** c** CRSwNP group, **d** CRSsNP group, **e** CRSwNPwAR group and** f** CRSsNPwAR group. Red arrows represent EOS cells (original magnification × 400, *p < 0.05, **p < 0.001). Bar 20 µm

**Fig. 2:**
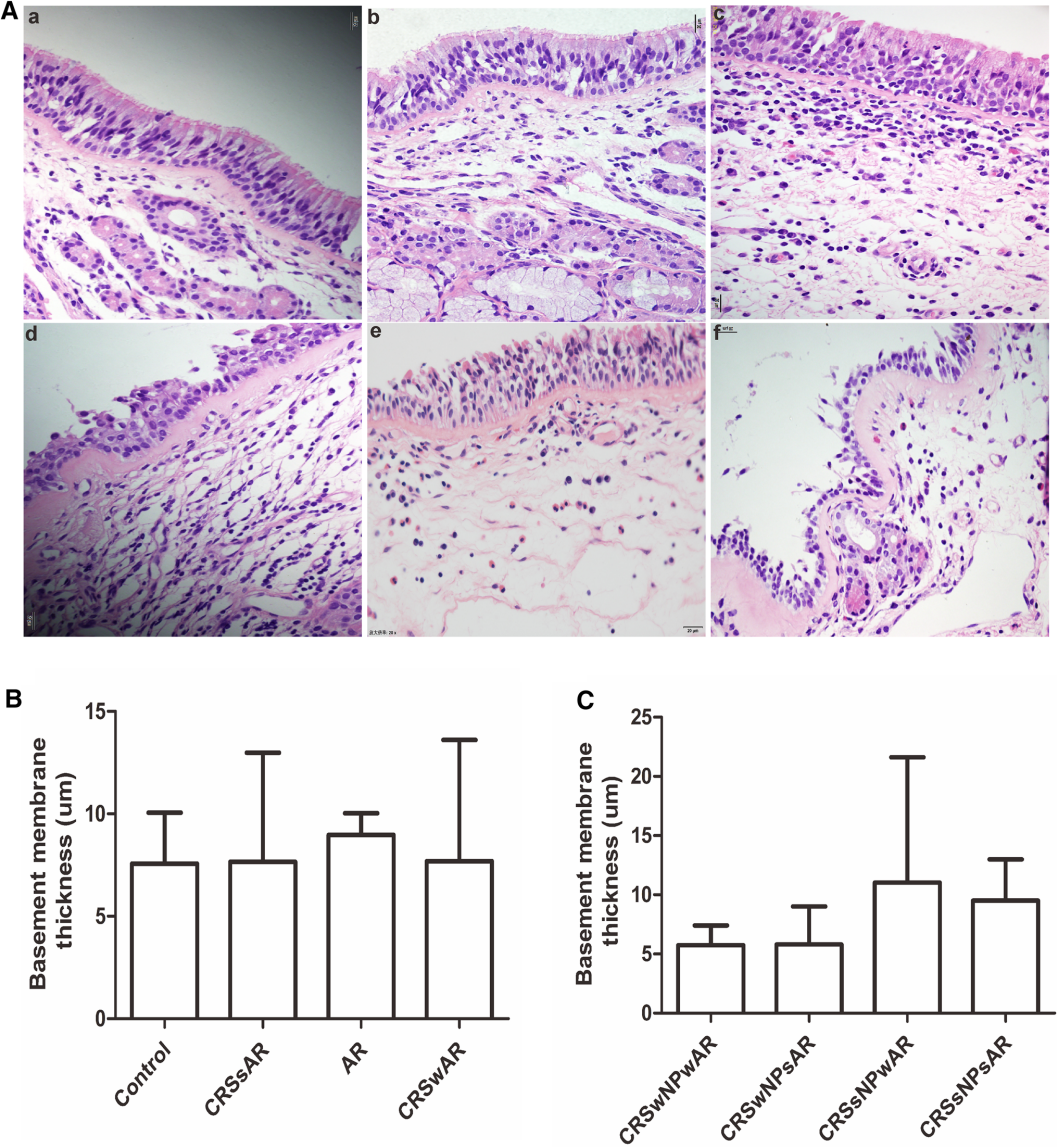
**A** Basement membrane thickness was examined by HE staining. **B**, **C** Quantification of basement membrane thickness in the nasal mucosa.** a** control group,** b** AR group,** c** CRSwNP group,** d** CRSsNP group,** e** CRSwNPwAR group and **f** CRSsNPwAR group. (original magnification × 400, *p < 0.05, **p < 0.001). Bar 20 µm

**Fig. 3:**
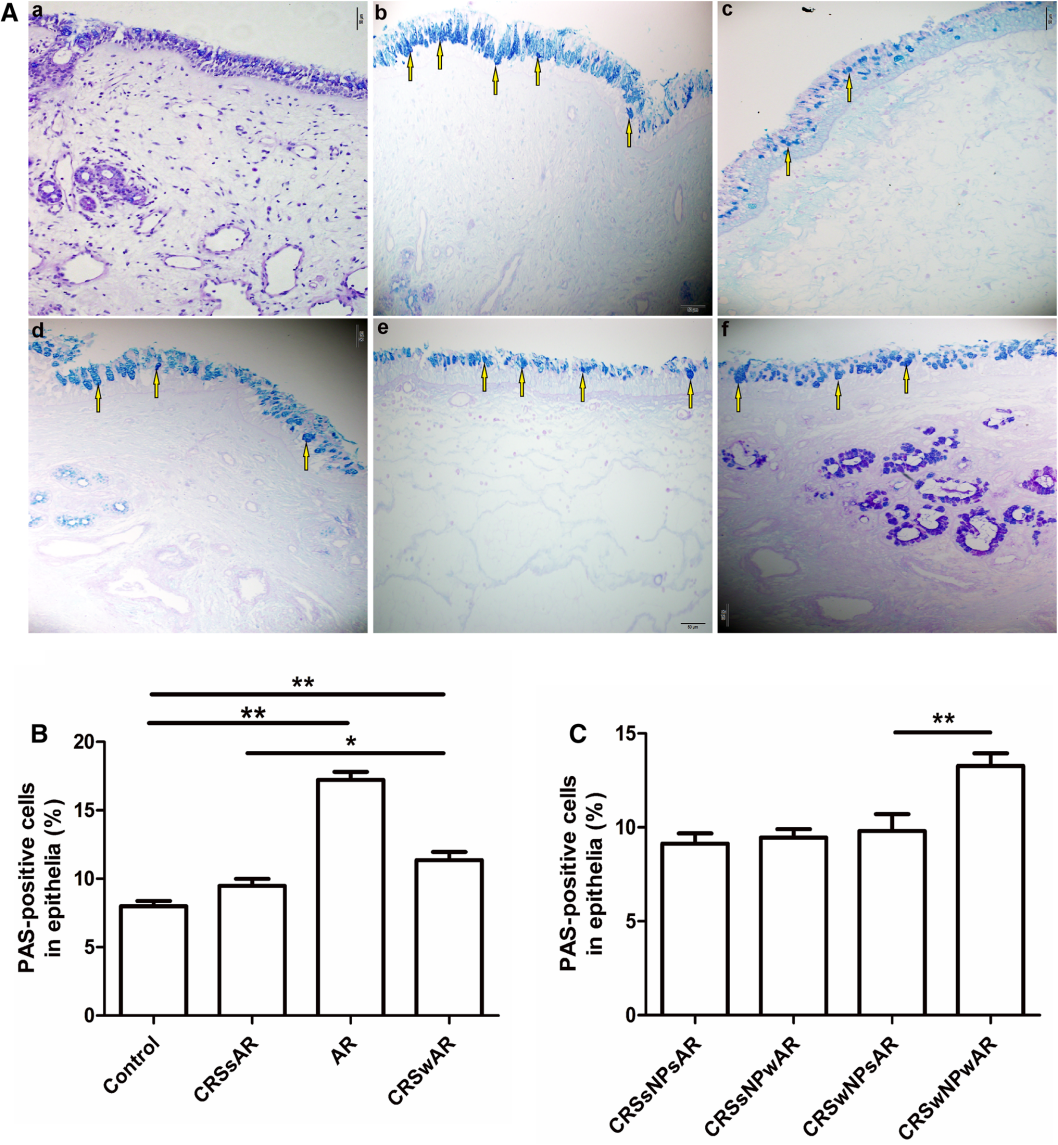
**A** PAS-positive cells in epithelia was examined by PAS staining. **B**, **C** PAS quantification of PAS staining in epithelial of nasal mucosa.** a** control group,** b** AR group,** c** CRSwNP group,** d** CRSsNP group,** e** CRSwNPwAR group and **f** CRSsNPwAR group. Yellow arrows represent PAS-positive cells (original magnification × 200, *p < 0.05, **p < 0.001). Bar 50 µm

**Fig. 4:**
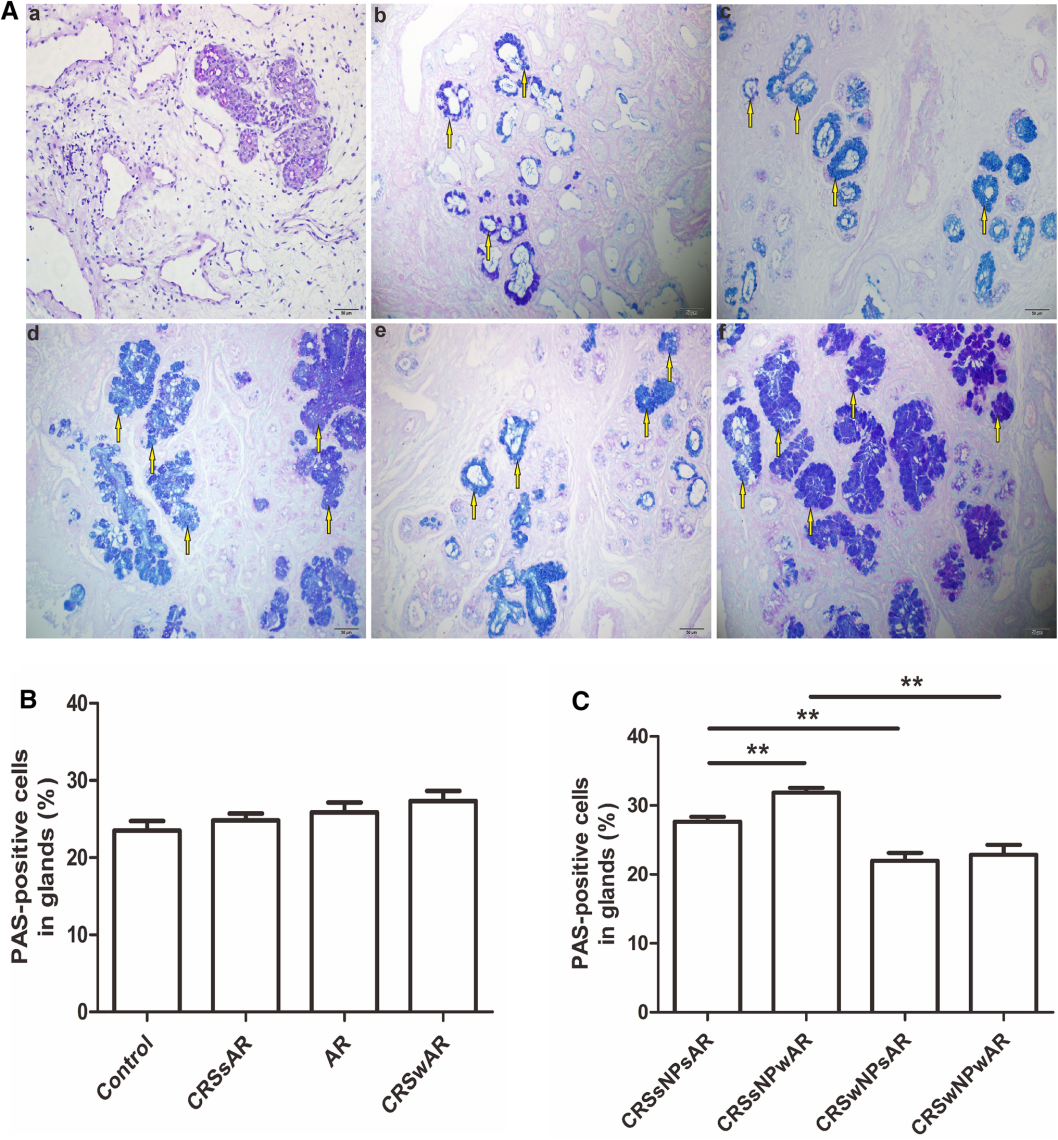
**A** Positive cells in glands was examined by PAS staining. **B**, **C** Quantification of PAS staining in glands of nasal mucosa.** a** control group,** b** AR group,** c** CRSwNP group,** d** CRSsNP group, **e** CRSwNPwAR group and **f** CRSsNPwAR group. Yellow arrows represent PAS-positive cells (original magnification × 200, *p < 0.05, **p < 0.001). Bar 50 µm

**Fig. 5:**
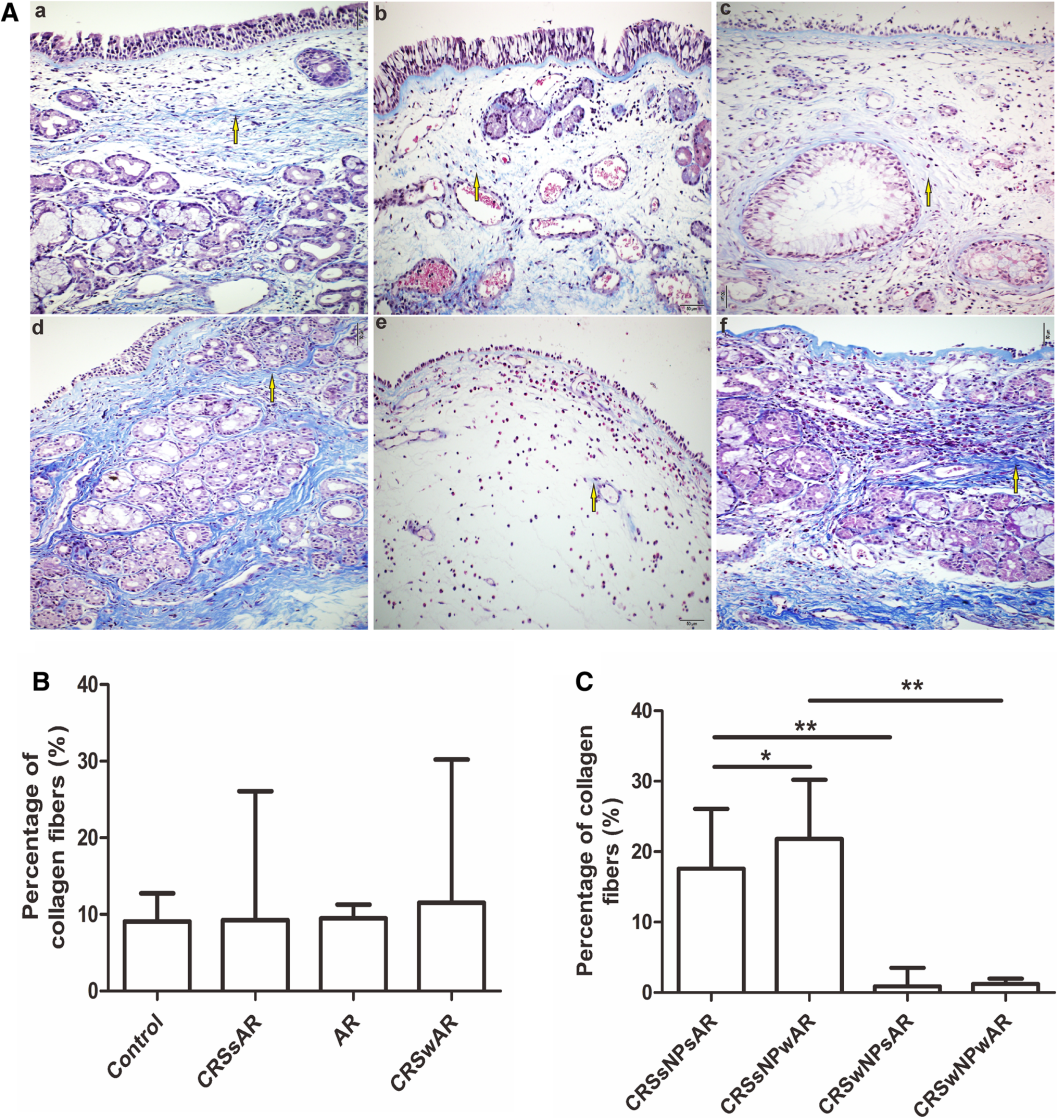
**A** Collagenous fibers was stained by Masson staining. **B**, **C** Quantification of collagen fibers in the nasal mucosa.** a** control group,** b** AR group,** c** CRSwNP group,** d** CRSsNP group,** e** CRSwNPwAR group and **f** CRSsNPwAR group. Yellow arrows represent collagenous fibers. (original magnification × 200, *p < 0.05, **p < 0.001). Bar 50 µm

The HE staining showed that the number of EOS in CRSwAR group was significantly higher compared to the CRSsAR group (164.2 ± 144.4 and 58.89 ± 65.68, respectively). Moreover, the number of EOS in CRSwNPwAR group was significantly higher compared to the CRSwNPsAR group(300.90 ± 49.30 and 113.60 ± 49.44, respectively).And, the number of EOS in the CRSsNPwAR group was significantly higher compared to the CRSsNPsAR group (27.41 ± 4.18 and 4.19 ± 3.92, respectively)(Fig. 1). No significant difference was observed in basement membrane thickness among different groups (Fig. 2). PAS staining showed that increased goblet cell hyperplasia was observed in CRSwAR group compared to CRSsAR group (11.37 ± 2.633 and 9.46 ± 2.32, respectively)(Fig. 3). And, the number of positive goblet cells in CRSwNPwAR group was significantly higher compared to the CRSwNPsAR group (13.27 ± 2.14 and 9.80 ± 2.86, respectively). Moreover, increased submucosal glands were observed in the CRSsNPwAR group compared to CRSsNPsAR group (31.86 ± 2.18 and 27.64 ± 2.24, respectively)(Fig. 4). Masson’s trichrome staining and quantitative analysis were applied to evaluate the content of collagen in the nasal mucosa. Quantitative analysis revealed that the content of collagen in the CRSsNPwAR group was significantly higher than the CRSsNPsAR group (21.82 ± 5.74 and 17.58 ± 4.48, respectively) (Fig. 5).

### Immunohistochemical analysis of VEGF-A and microvessel density

**Fig. 6:**
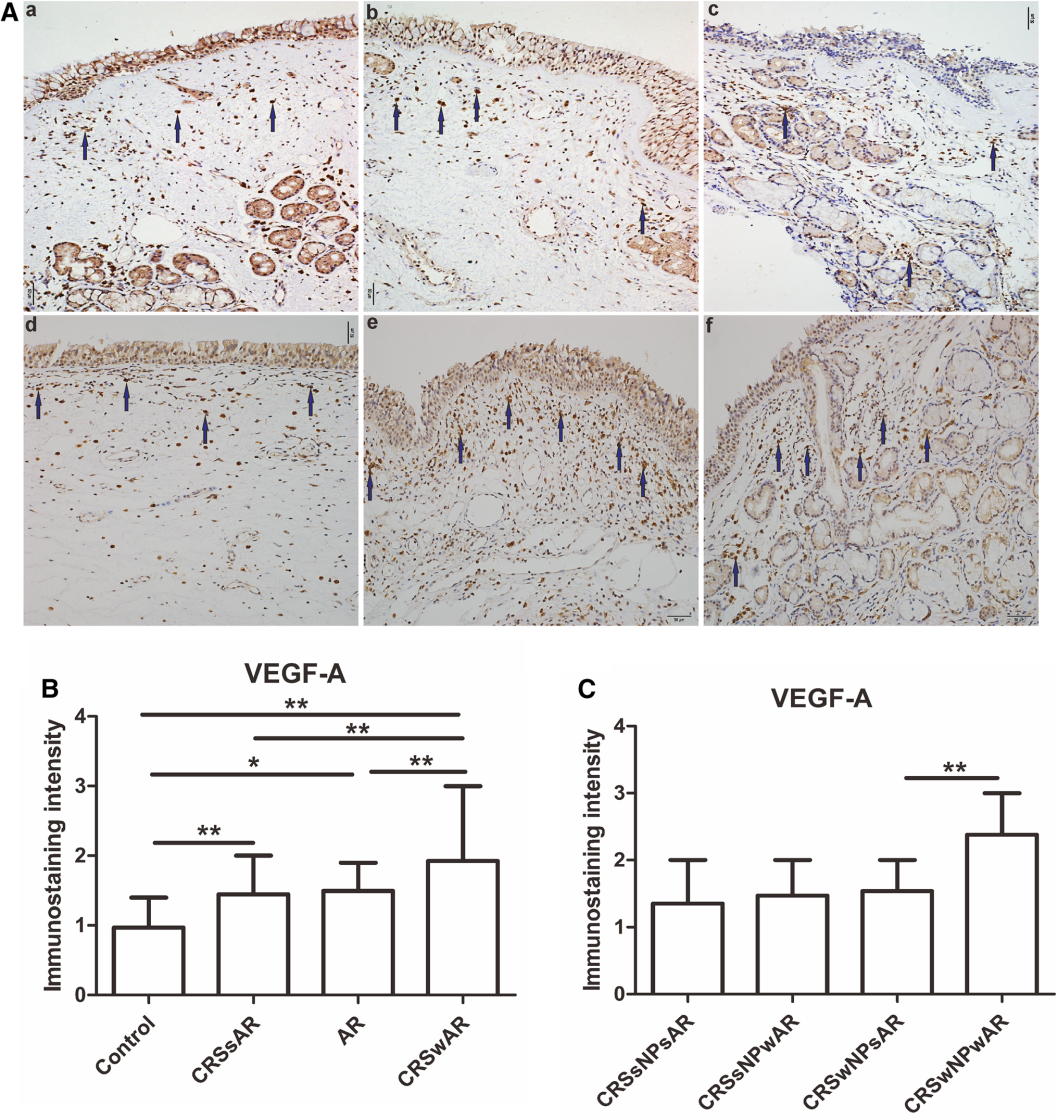
**A** Immunolabeling of VEGF-A in the nasal mucosa. **B**, **C** Quantification of immunostaining intensity of VEGF-A expression in the nasal mucosa.** a** control group,** b** AR group,** c** CRSwNP group,** d** CRSsNP group,** e** CRSwNPwAR group and** f** CRSsNPwAR group. Blue arrows represent VEGF-A positive cells (original magnification × 200, *p < 0.05, **p < 0.001). Bar 50 µm

To assess the effect of AR on nasal mucosa remodeling in CRS, VEGF-A was detected by IHC. The results revealed that the VEGF-A expression was significantly increased in CRSwAR group compared to CRSsAR group (1.92 ± 0.55 and 1.44 ± 0.37, respectively). The expression of VEGF-A in CRSwNPwAR group was higher compared to CRSwNPsAR group (2.38 ± 0.35 and 1.54 ± 0.39, respectively). However, no significant difference was observed between CRSsNPsAR group and CRSsNPwAR group (Fig. 6, *p* > 0.05).

**Fig. 7:**
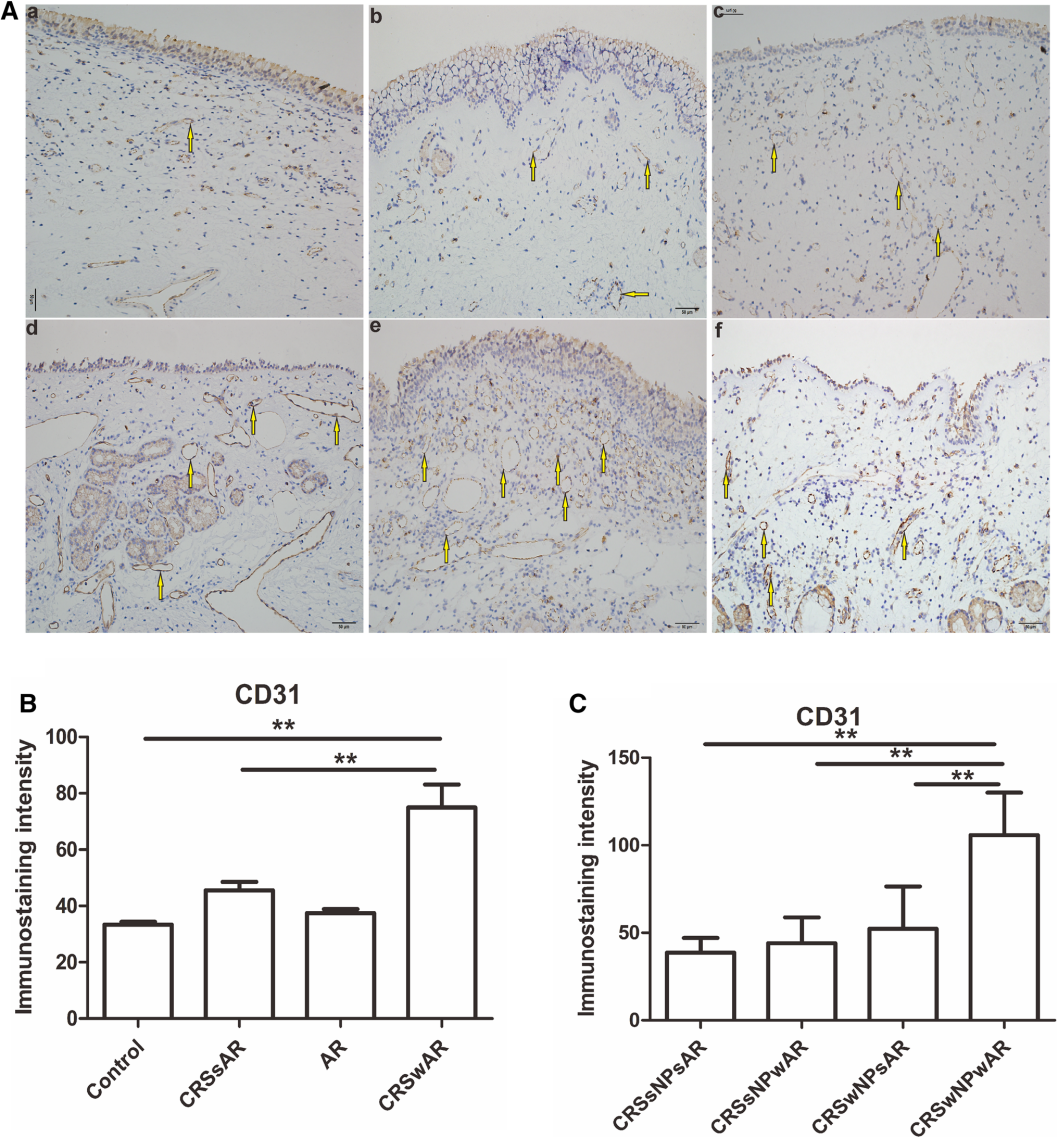
**A** Immunolabeling of CD31 in the nasal mucosa. **B**, **C** Quantification of immunostaining intensity of CD31 in the nasal mucosa.** a** control group,** b** AR group,** c** CRSwNP group,** d** CRSsNP group,** e** CRSwNPwAR group and** f** CRSsNPwAR group. Yellow arrows represent CD31-positive cells (original magnification × 200, *p < 0.05, **p < 0.001). Bar 50 µm

**Fig. 8:**
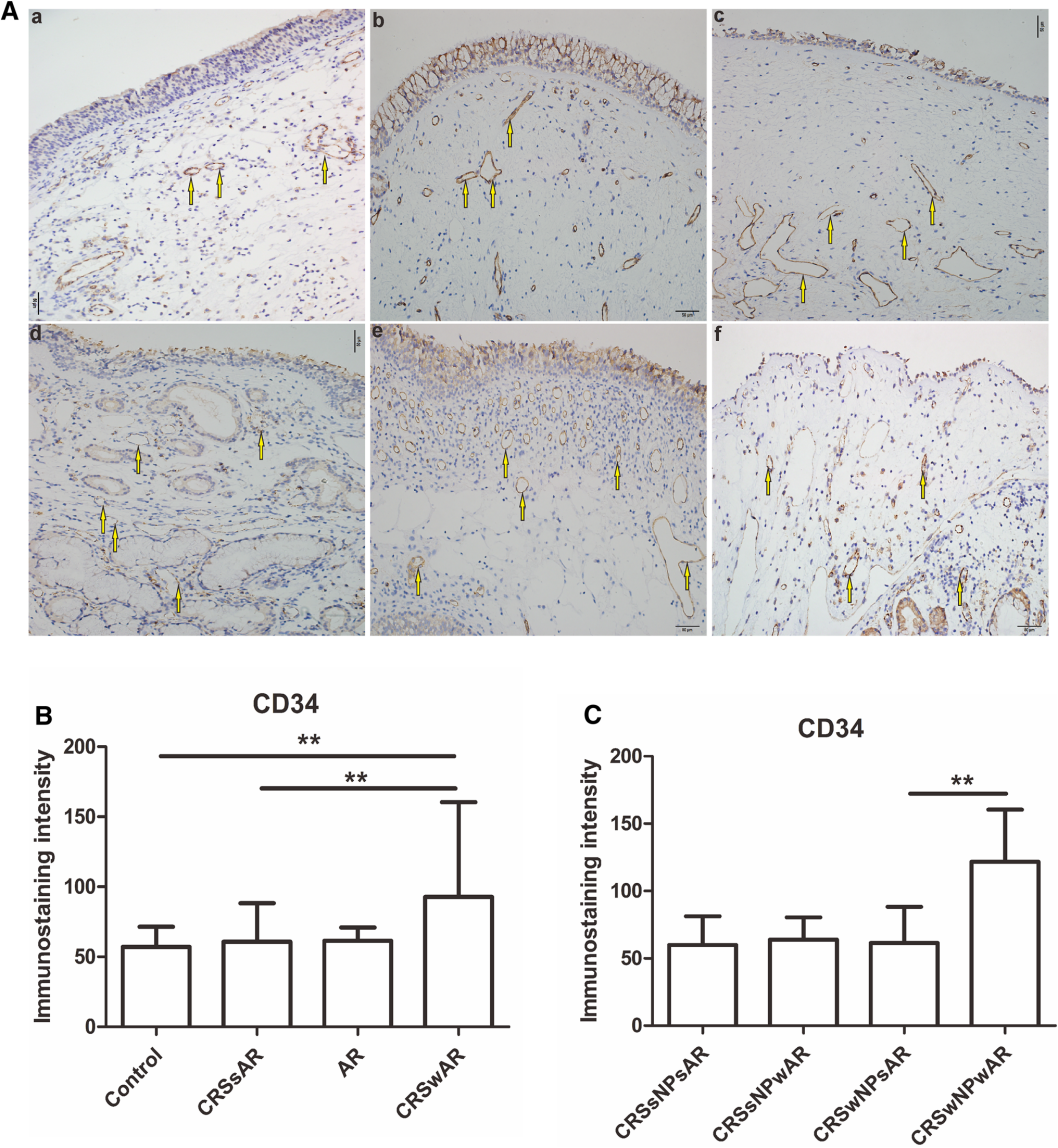
**A** Immunolabeling of CD34 in the nasal mucosa. **B**, **C** Quantification of immunostaining intensity of CD34 in the nasal mucosa.** a** control group,** b** AR group,** c** CRSwNP group,** d** CRSsNP group,** e** CRSwNPwAR group and** f** CRSsNPwAR group. Yellow arrows represent CD34-positive cells (original magnification × 200, *p < 0.05, **p < 0.001). Bar 50 µm

IHC of CD31 and CD34 were performed to detect microvessel angiogensis in the present study. As shown in Fig. 7, the density of CD31 was significantly higher in CRSwAR group compared to the CRSsAR group (92.83 ± 33.39 and 60.68 ± 10.12, respectively). Moreover, the density of CD31 in CRSwNPwAR group was higher compared to CRSwNPsAR group(105.90 ± 23.83 and 52.35 ± 14.39, respectively). However, no significant difference was observed between CRSsNPwAR group and CRSsNPsAR group. As shown in Fig. 8, the density of CD34 was significantly higher in CRSwAR group than the CRSsAR group (92.83 ± 33.39 and 60.68 ± 10.12, respectively). Moreover, the density of CD34 in CRSwNPwAR group was higher compared to CRSwNPsAR group (121.80 ± 20.98 and 61.47 ± 12.28, respectively). However, no significant difference was observed between the CRSsNPwAR group and CRSsNPsAR group.

### The expression of TGF-β1, MMP-7, MMP-9 and TIMP-1

**Fig. 9:**
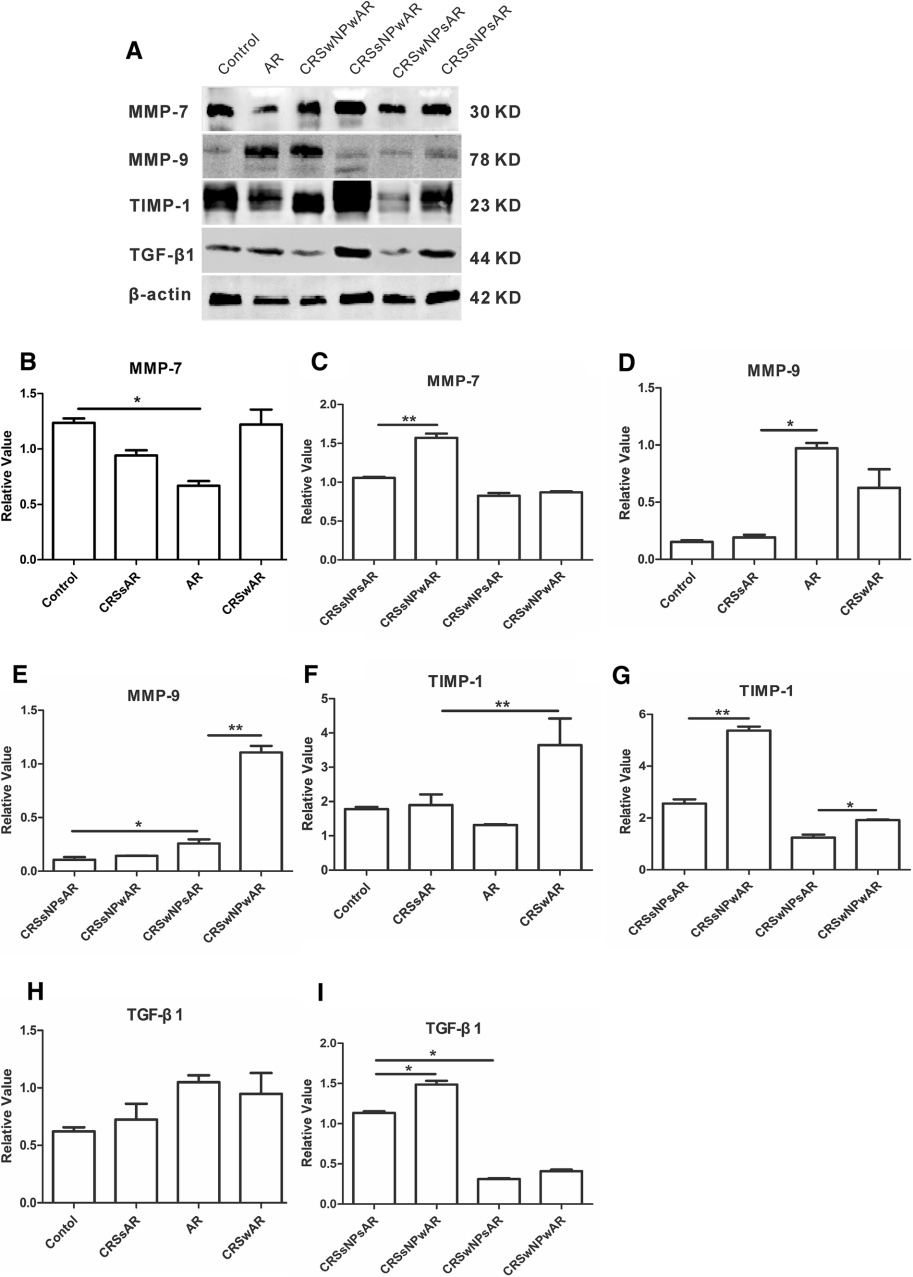
**a** Western blotting analysis of MMP-7, MMP-9, TIMP-1 and TGF-β1 in control group, AR group, CRSwNP group, CRSsNP group, CRSwNPwAR group and CRSsNPwAR group. **b**–**i** Semi-quantitative analysis of MMP-7, MMP-9, TIMP-1 and TGF-β1 (*p < 0.05, **p < 0.001)

We investigated the expression of TGF-β1, MMP-7, MMP-9 and TIMP-1 by Western blotting. As shown in Fig. 9, the expression of MMP-9 was significantly higher in CRSwNPwAR group compared to CRSwNPsAR group (1.10 ± 0.13 and 0.25 ± 0.08, respectively). However, no significant difference was observed between CRSsNPwAR group and CRSsNPsAR group. The expression of MMP-7 and TGF-β1 in CRSsNPwAR group was higher compared to the CRSsNPsAR group (1.57 ± 0.11 and 1.05 ± 0.02 for MMP-7 and 1.48 ± 0.10 and 1.13 ± 0.04 for TGF-β1). However, the expression of MMP-7 and TGF-β1 in the CRSwNPsAR group was not significantly different from those in the CRSwNPwAR group. The expression of TIMP-1 was significantly higher in CRSwAR patients compared to CRSsAR patients (3.64 ± 1.90 and 1.89 ± 0.75, respectively). And, the expression of TIMP-1 was significantly higher in CRSsNPwAR patients compared to CRSsNPsAR patients(5.37 ± 0.27 and 2.55 ± 0.27, respectively). Besides, the expression of TIMP-1 was also significantly higher in CRSwNPwAR patients compared to the CRSwNPsAR patients (1.97 ± 0.03 and 1.24 ± 0.20, respectively).

## Discussion

Tissue modeling is a process that occurs throughout the body in relation to inflammation or mechanical injury [16]. It is a dynamic process of excessive extracellular matrix (ECM) production and protease inhibitor-controlled degradation [17]. The airway remodeling was first described in the lower airway in asthma. Recently, a growing numbers of studies investigated tissue remodeling in CRS and found that CRS is also characterized by mucosa remodeling [3]. It was reported that mucosal hypertrophy, basement membrane thickening, fibrosis, subepithelial collagen deposition and angiogenesis are common forms of remodeling in CRS [3]. These tissue remodeling changes in CRS could affect normal sinonasal physiology, increase duration of symptoms and enhance surgical difficulty [18]. Moreover, the increased expression of fibroblasts in CRSwNP patients was correlated with subjective disease severity [19].

Airway diseases such as asthma, chronic obstructive pulmonary disorder (COPD) and CRS are all characterized by both inflammation and remodeling. Researchers found a close relationship between inflammation and remodeling [20, 21]. Traditionally, chronic inflammation is believed to be the cause of remodeling. However, new evidence suggests that remodeling is an active primary process that could occur with ongoing inflammation and contribute to inflammation [21]. Inflammatory cells such as EOS and neutrophil cells are proved to be associated with mucosa remodeling in CRS. Sampson et al. [22] showed that EOS could induce epithelium and ECM damage by releasing toxic proteins, lipid mediators and reactive oxygen species and subsequently lead to edema formation. Whereas, neutrophils exerts its role in remodeling by secreting remodeling mediators, such as MMPs and TGF-β in non-eosinophilic CRS [23]. AR is a Th2-skewed inflammation with high levels of IL-4, IL-13 and IL-5, and elevated inflammatory cells infiltration including EOS and neutrophils [24]. Studies suggested that AR could affect the pathogenesis of CRS and the effects could be different between distinct races. A study investigated in America demonstrated that AR was associated with decreased odds of undergoing endoscopic sinus surgery in CRS patients [25]. While AR was proved to be a risk factor for CRS in studies conducted in South Korea [26]. This discrepancy might be caused by the fact that eosinophilic CRS was the most common type of CRSwNP in Western countries, whereas neutrophilic inflammation was more common in cases of CRSwNP in Asia [27]. However, little literature investigated the effect of AR on mucosa remodeling in CRS. We hypothesize that AR, as an inflammatory disorder, may promote the disease severity, prolong the duration of symptoms of CRS by regulating the mucosa remodeling. We determined the effect of AR on mucosa remodeling in CRS by assessing the tissue structures, including epithelium, submucosal glands, basement membrane thickness, collagen deposition and EOS infiltration. In the present study, we showed that increased numbers of EOS and higher content of collagen deposition were observed in CRSwAR patients compared to CRSsAR patients. These results suggested that AR could enhance the remodeling process in CRS.

VEGF is widely recognized as a major proangiogenic factor involved in the process of neovascularization and vascular leakage through VEGF receptor-mediated pathways in tissue remodeling [28]. Previous study [29] demonstrated high levels of VEGF in nasal polyps enhanced proliferation by activating VEGF receptors and downstream pathways, subsequently promoting polyps formation. VEGF is a family of homodimeric proteins that consisted by six members, including VEGF-A, VEGF-B, VEGF-C, VEGF-D, VEGF-E and placenta growth factor [30]. VEGF-A, the most studied VEGF family member, was shown to play a pivotal role in neovascularization regulation [31]. CD34, a cell surface sialomucin, is involved in a variety of pathophysiologic processes, including cell adhesion, inflammatory cell recruitment and angiogenesis. CD31, also described as PECAM-1 (platelet endothelial cell adhesion molecule 1), plays a critical role in angiogenesis [32].CD31 and CD34 are endothelial antigens that have been used to evaluate the density of microvessel as direct markers of neoangiogenesis [33]. In the present study, CD31, CD34 and VEGF were analyzed together to support the effect of AR on angiogenesis. We observed that the expression levels of VEGF-A, CD31 and CD34 were significantly increased in the CRSwAR group compared to the CRSsAR group. In addition, the expression of VEGF-A and microvessel density were significantly higher in the CRSwNPwAR group compared to the CRSwNPsAR group. Matsune et al. [34] reported that nasal secretion VEGF levels were higher in AR subjects compared to the controls. Hypersecreted VEGF in nasal cavity promoted vascular permeability and mucosal edema. The elevated expression of VEGF, CD31 and CD34 may contribute to NP formation and congestion syndrome in patients with CRSwAR, especially in patients with CRSwNPwAR. Thus, these findings together indicated that AR could enhance remodeling by promoting angiogenesis in CRS.

MMPs is a family of proteolytic enzymes that implicated tissue remodeling, proliferation and migration by breaking down ECM components, facilitating epithelial cell migration and modulating the state of cell–matrix and cell–cell interactions [35]. MMPs and their tissue inhibitors (TIMPs) are considered to play important roles in nasal polyp formation [36]. MMP-9, secreted by epithelial cells, EOS, mast cells and activated neutrophils, plays a critical role in tissue remodeling in CRS through decomposing the components of ECM. A previous study [37] showed that MMP-9 and MMP-7 were significantly increased in CRSwNP patients compared to controls. Higher levels of MMP-9 and low levels of TIMP-1 were observed in patients with CRSwNP. Meanwhile, MMPs were also found to participate in regulating inflammatory cells infiltration and microvascular permeability in allergic disorders including AR and asthma [36, 38]. Increased expression of MMP-9 and ratio of MMP-9/TIMP-1 were observed in AR. And the imbalance of MMPs and TIMPs may play a role in AR by contributing to the migration of inflammatory cells (e.g., EOS and mast cells) to the nasal mucosa. In the present study, we showed that MMP-9 expression and MMP-9/TIMP-1 ratio were significantly increased in the CRSwNPwAR group compared to the CRSwNPsAR group. Thus, these data indicated that elevated ratio of MMP-9/TIMP-1 enhances the nasal polyps formation in CRS by recruiting EOS infiltration and decomposing the components of ECM. Besides, MMP-9 was found to be significantly correlated with healing quality after surgery and could be lower expressed after corticosteroid treatment in patients with CRSsNP [39]. This suggested that MMP-9 could be therapeutic targets for CRS. We speculated that higher expression of MMP-9 could contribute to longer surgery recovery time in patients with CRSwAR. Yang et al. [40] showed that MMP-7 was significantly increased in EOS CRSwNP compared to non-Eos CRSwNP. However, our data showed that MMP-7 expression was higher in CRSsNPwAR group compared to CRSsNPsAR group and no significant difference was observed between CRSwNPsAR group and CRSwNPwAR group. This discrepancy may arise because that CRS subtypes differ in distinct races. Further studies are still required to clarify the role of MMPs and TIMPs in CRS.

TGF-β, a multifunctional cytokine that can regulate cell proliferation, differentiation, and migration, is associated with a variety of inflammatory disorders [41]. New evidence demonstrated that TGF-β also plays an important role in tissue remodeling processes via promoting the differentiation of fibroblasts into myofibroblasts and enhancing collagen synthesis [12]. Dysregulation of TGF-β activation and expression has been shown to be involved in the pathogenesis of a variety of airway diseases related to remodeling, including asthma and CRS. A clinical study [42], revealed that patients with CRSsNP had higher levels of TGF-β compared to healthy individuals, whereas patients with CRSwNP had lower TGF-β expression. Furthermore, Nicholas et al. [43] demonstrated that decreased TGF-β1 expression could be related to the edema formation in CRSwNP, whereas increased TGF-β1 expression could play a critical role in the excessive tissue repair and fibrosis formation in CRSsNP. In coincidence with the previous study, our results revealed that the expression of TGF-β1 in CRSsNPwAR group was higher compared to the CRSsNPsAR group. Therefore, it is reasonable to speculate that AR could enhance remodeling process by promoting the expression of TGF-β1 in CRSsNP.

A limitation of this study is that we did not investigate the correlation between severity of AR and remodeling degree in CRS. Further studies on relationship between severity of AR and remodeling in CRS are needed.

## Conclusion

In conclusion, our results suggest that AR could enhance the remodeling process in CRS. Moreover, we found AR had different effects on CRSwNP and CRSsNP. For AR enhanced goblet cell hyperplasia, as well as VEGF-A, microvessel density, and MMP-9 expression in CRSwNP and promoted the expression of collagen fibers, TGF-β1 and MMP-7 in CRSsNP.

## Abbreviations

CRS: Chronic rhinosinusitis
CRSwNP: Chronic rhinosinusitis with nasal polyps
CRSsNP: Chronic rhinosinusitis without nasal polyps
IHC: Immunohistochemical
